# 2711. Viral encephalitis after chimeric antigen receptor (CAR)-modified T-cell therapy: A Retrospective Cohort Study

**DOI:** 10.1093/ofid/ofad500.2322

**Published:** 2023-11-27

**Authors:** Eleftheria Kampouri, Erika Kiem, Alythia Vo, Winnie L Liu, Clementine Chalal, Ryan S Basom, Chris Davis, Cameron J Turtle, Mazyar Shadman, Brian Till, Ryan D Cassaday, Jordan Gauthier, Aude G Chapuis, David G Maloney, Elizabeth M Krantz, Joshua A Hill

**Affiliations:** Fred Hutchinson Cancer Center, Seattle, WA; Fred Hutchinson Cancer Center, Seattle, WA; Fred Hutchinson Cancer Center, Seattle, WA; Fred Hutchinson Cancer Center, Seattle, WA; Fred Hutchinson Cancer Center, Seattle, WA; Fred Hutchinson Cancer Center, Seattle, WA; Fred Hutchinson Cancer Research Center, Seattle, Washington; Fred Hutchinson Cancer Research Center / Seattle Care Cancer Alliance / University of Washington School of Medicine, Seattle, WA, Seattle, Washington; Fred Hutchinson Cancer Center, Seattle, WA; Fred Hutchinson Cancer Center, Seattle, WA; Fred Hutchinson Cancer Center, Seattle, WA; Fred Hutchinson Cancer Center, Seattle, WA; Fred Hutchinson Cancer Center, Seattle, WA; Fred Hutchinson Cancer Research Center / Seattle Care Cancer Alliance / University of Washington, Seattle, WA, Seattle, Washington; Fred Hutch Cancer Center, Seattle, Washington; Fred Hutchinson Cancer Center; University of Washington, Seattle, Washington

## Abstract

**Background:**

Central nervous system (CNS) symptoms are frequent after chimeric antigen receptor T-cell therapy (CARTx) and usually attributed to immune effector cell-associated neurotoxicity syndrome (ICANS). Cerebrospinal fluid (CSF) testing to rule out CNS infection is not routinely performed, although ICANS and viral encephalitis have overlapping presentations. Reports of viral encephalitis after CARTx are increasing, but no cohort studies have been performed to understand its epidemiology.

**Methods:**

This retrospective cohort study included all adults receiving first CARTx for B-cell malignancies at Fred Hutch Cancer Center from July 2013 to December 2022. We computed the cumulative incidence of CSF testing for viruses within 56 days after CARTx.

**Results:**

626 patients received CARTx during the study period; 552 (88.2%) received CD19 or CD20-targeted CARTx and 74 (11.8%) BCMA-targeted CARTx. Median age was 60.4 (IQR: 50.9–67.6) years, 232 (37.1%) identified as female, and 216 (34.5%) received prior hematopoietic cell transplantation. Thirty-four patients had CSF tested for viruses with PCR in the context of CNS symptoms for a cumulative incidence of 5.7% (95% confidence interval, 4.0–7.8) (**Figure 1**). ICANS was diagnosed in 29 (85.3%) of these 34 patients (ICANS grade ≥ 3 in 14), and 26 patients received immunosuppressive treatment. In the remaining five tested patients, the cause of CNS symptoms was vascular (n=1), CNS progressive disease (n=2), metabolic encephalopathy (n=1) or unknown (n=1). CSF testing practices evolved overtime, and testing was performed more frequently before 2018 (**Figure 2**); after 2018, all tested patients had ICANS grade ≥ 3. Four patients had a positive CSF PCR test for EBV (n=3) occurring 8–39 days post-CARTx or HHV-6 (n=1) on day 32 post-CARTx. EBV detection in CSF was low-level, of unclear clinical significance, and not treated; all three patients were treated for ICANS. The patient with HHV-6 had low-level detection in CSF and blood; HHV-6 encephalitis was considered, but no antiviral therapy was administered, and symptoms resolved with treatment for ICANS grade 4.

**Figure:**
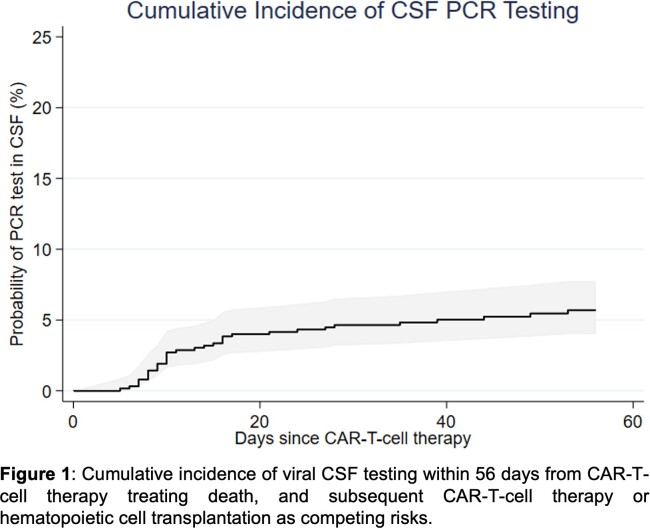

**Figure:**
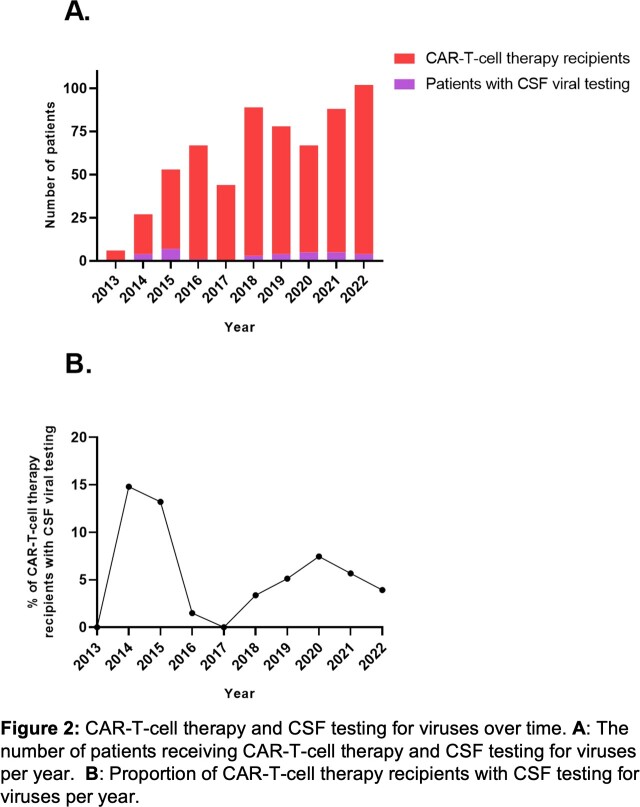

**Conclusion:**

CSF testing for infection early after CARTx was rarely performed, and the incidence of viral encephalitis appears to be low in this population.

**Disclosures:**

**Cameron J. Turtle, MBBS, PhD**, Allogene: Advisor/Consultant|Allogene: Ad hoc advisory boards/consulting|ArsenalBio: Advisor/Consultant|ArsenalBio: Scientific Advisory Board, stock options|ArsenalBio: Stocks/Bonds|Caribou Biosciences: Advisor/Consultant|Caribou Biosciences: Stocks/Bonds|Century Therapeutics: Advisor/Consultant|Century Therapeutics: Ad hoc advisory boards/consulting|Eureka Therapeutics: Stocks/Bonds|Fred Hutchinson Cancer Center: CJT has the right to receive payment from Fred Hutchinson Cancer Center as an inventor on patents related to CAR-T-cell therapy|Juno Therapeutics/BMS: Grant/Research Support|Kyverna: DSMB member|Legend Bio: Advisor/Consultant|Legend Bio: Ad hoc advisory boards/consulting|Myeloid Therapeutics: Advisor/Consultant|Myeloid Therapeutics: Scientific Advisory Board, stock options|Myeloid Therapeutics: Stocks/Bonds|Nektar Therapeutics: Advisor/Consultant|Nektar Therapeutics: Grant/Research Support|Prescient Therapeutics: Advisor/Consultant|Prescient Therapeutics: Ad hoc advisory boards/consulting|Sobi: Advisor/Consultant|Sobi: Ad hoc advisory boards/consulting|Syncopation Life Sciences: Advisor/Consultant|Syncopation Life Sciences: Ad hoc advisory boards/consulting|T-CURX: Advisor/Consultant|T-CURX: Scientific advisory board **Mazyar Shadman, MD**, AbbVie: Advisor/Consultant|AbbVie: Grant/Research Support|ADC therapeutics: Advisor/Consultant|AstraZeneca: Advisor/Consultant|AstraZeneca: Grant/Research Support|BeiGene: Advisor/Consultant|BeiGene: Grant/Research Support|BMS: Advisor/Consultant|BMS: Grant/Research Support|Eli Lilly: Advisor/Consultant|Fate Therapeutics: Advisor/Consultant|Genentech: Advisor/Consultant|Genentech: Grant/Research Support|Genmab: Advisor/Consultant|Genmab: Grant/Research Support|Kite: Advisor/Consultant|MorphoSys/Incyte: Advisor/Consultant|MorphoSys/Incyte: Grant/Research Support|Mustang Bio: Advisor/Consultant|Mustang Bio: Grant/Research Support|Nurix and MEI Pharma: Advisor/Consultant|Pharmacyclics: Advisor/Consultant|Pharmacyclics: Grant/Research Support|Regeneron: Advisor/Consultant|TG Therapeutics: Grant/Research Support|Vincerx: Grant/Research Support **Brian Till, MD**, Juno Therapeutics/BMS: Grant/Research Support|Mustang Bio: Grant/Research Support|Mustang Bio: patents/royalties|Mustang Bio: Consulting|Proteios Technology: Advisor/Consultant **Ryan D. Cassaday, MD**, Autolus: Independent response review committee|Kite/Gilead: Grant/Research Support|Kite/Gilead: Honoraria **Jordan Gauthier, MD**, Angiocrine Bioscience: Grant/Research Support|Celgene (a BMS company): Grant/Research Support|Century Therapeutics: Independent Data Review Committee|Janssen: Advisor/Consultant|Janssen: Honoraria|Juno Therapeutics (a BMS company): Grant/Research Support|Kite Pharma: Advisor/Consultant|Kite Pharma: Honoraria|Legend Biotech: Advisor/Consultant|Legend Biotech: Honoraria|MorphoSys: Advisor/Consultant|MorphoSys: Honoraria|Sobi: Advisor/Consultant|Sobi: Grant/Research Support|Sobi: Honoraria **David G. Maloney, MD, PhD**, A2 Biotherapeutics: Membership with compensation: member of scientific advisory board|BMS: Advisor/Consultant|BMS: Grant/Research Support|BMS: Honoraria|BMS: Member (compensation): JCAR017 EAP-001 Safety Review Committee, CLL Strategic Council. Without compensation: JCAR017-BCM03 Scientific Steering Committ|Celgene: Advisor/Consultant|Celgene: Grant/Research Support|Celgene: Honoraria|Chimeric Therapeutics: Membership with compensation: member of scientific advisory board|Genentech: Advisor/Consultant|Genentech: Honoraria|Genentech: Membership with compensation: member and chair of the Lymphoma Steering Committee|Gilead Sciences: Membership with compensation: Member Scientific Review Committee, Research Scholars Program in Hematologic Malignancies|ImmPACT Bio: Membership with compensation: Member, Clinical Advisory Board, CD19/CD20 bi-specific CAR-T Cell Therapy Program|Interius: Membership with compensation: member clinical advisory board|Juno Therapeutics: Advisor/Consultant|Juno Therapeutics: Grant/Research Support|Juno Therapeutics: Honoraria|Juno Therapeutics/BMS: Dr. Maloney has the rights to royalties from Fred Hutchinson Cancer Center for patents licensed to Juno Therapeutics/BMS|Kite: Advisor/Consultant|Kite: Grant/Research Support|Kite: Honoraria|Legend Biotech: Grant/Research Support|Navan Technologies: Membership with compensation: member of scientific advisory board **Joshua A. Hill, MD**, Allovir: Advisor/Consultant|Allovir: Grant/Research Support|Century Therapeutics: Advisor/Consultant|Covance/CSL: Advisor/Consultant|Deverra: Grant/Research Support|Eversana Life Science Services, LLC: Advisor/Consultant|GeoVax: Grant/Research Support|Gilead: Advisor/Consultant|Gilead: Grant/Research Support|Karius: Advisor/Consultant|Karius: Grant/Research Support|Merck: Grant/Research Support|Moderna DSMB: Advisor/Consultant|Octapharma AG: Advisor/Consultant|OptumHealth: Advisor/Consultant|Oxford Immunotec: Grant/Research Support|Pfizer (previously Amplyx/Medpace): Advisor/Consultant|Senti BioSciences, Inc: Advisor/Consultant|Symbio: Advisor/Consultant|Takeda: Advisor/Consultant|Takeda: Grant/Research Support|Up-to-Date: Advisor/Consultant

